# Urinary Tract Infections: Molecular Determinants of Uropathogenicity and Their Translation into Contemporary Clinical Practice

**DOI:** 10.3390/ijms27188108

**Published:** 2026-09-11

**Authors:** Raul-Lucian Ene, Roxana Popescu, Aurica Elisabeta Cobec, Melinda Kali, Ileana-Adriana Ene, Daliborca Cristina Vlad, Peter Seropian, Ionut Marcel Cobec

**Affiliations:** 1 Doctoral School, Faculty of Medicine, “Victor Babes” University of Medicine and Pharmacy Timisoara, 300041 Timisoara, Romania; 2 Department of Urology, Emergency Hospital Petrosani, 332019 Petrosani, Romania; 3 ANAPATMOL Research Center, Faculty of Medicine, “Victor Babes” University of Medicine and Pharmacy Timisoara, 300041 Timisoara, Romania; 4 Department of Cell and Molecular Biology, “Victor Babes” University of Medicine and Pharmacy Timisoara, 300041 Timisoara, Romania; 5 Clinic of Internal Medicine-Cardiology, Klinikum Freudenstadt, 72250 Freudenstadt, Germany; 6 Diaverum Petroșani Nephrology and Dialysis Medical Center, 332019 Petrosani, Romania; 7 Department of Pharmacology, Faculty of Medicine, “Victor Babes” University of Medicine and Pharmacy Timisoara, 300041 Timisoara, Romania; 8 Clinic of Obstetrics and Gynecology, Klinikum Freudenstadt, 72250 Freudenstadt, Germany; 9 Department of Obstetrics and Gynecology, Faculty of Medicine, Medical Center-University of Freiburg, 79106 Freiburg, Germany

**Keywords:** urinary tract infections, Uropathogenic *Escherichia coli* (UPEC), urinary microbiome, antimicrobial resistance, ESKAPE pathogens, intracellular bacterial communities, complicated UTI, immunoprophylaxis, bacteriophage therapy

## Abstract

Urinary tract infections (UTIs) represent a major global health burden, traditionally defined as microbial invasion of a sterile urinary tract, but now increasingly understood as a state of microbial dysbiosis involving disruption of the urinary microbiome. This review aims to synthesize current knowledge on the molecular mechanisms, etiological agents, clinical classification, and emerging therapeutic strategies in UTIs. The analysis integrates recent advances in microbiome research, molecular pathogenesis, and clinical guidelines. Uropathogenic *Escherichia coli* (UPEC) remains the predominant pathogen, utilizing virulence factors such as adhesins and intracellular bacterial community formation to establish persistent infection, while other organisms including *Klebsiella pneumoniae*, *Proteus mirabilis*, and *Enterococcus faecalis* contribute to disease complexity. The emergence of multidrug-resistant organisms, particularly among ESKAPE pathogens (*Enterococcus faecium*, *Staphylococcus aureus*, *Klebsiella pneumoniae*, *Acinetobacter baumannii*, *Pseudomonas aeruginosa*, and *Enterobacter species*), poses significant therapeutic challenges through mechanisms such as β-lactamase production, efflux pumps, and biofilm formation. Clinically, evolving classification systems emphasize infection localization rather than host factors, improving diagnostic and therapeutic precision. Diagnostic strategies rely on clinical assessment, urinalysis, and urine culture, while treatment increasingly incorporates antimicrobial stewardship principles. Emerging approaches, including immunoprophylaxis, bacteriophage therapy, and microbiome-targeted interventions, demonstrate promising results. In conclusion, UTIs are complex, multifactorial diseases requiring integrated molecular, clinical, and therapeutic approaches, with future advancements likely driven by precision medicine and artificial intelligence.

## 1. Introduction

Historically, UTIs were clinically described as not only the presence but also the multiplication of pathogenic microorganisms within the ostensibly “sterile” environment of the urinary system, encompassing the kidneys, ureters, bladder, and urethra. However, recent advancements in next-generation sequencing (NGS) and expanded quantitative urine culture (EQUC) have fundamentally dismantled the “sterile bladder” paradigm. It is now well established that the healthy human urinary tract harbours a complex, resident microbial community also known as the urinary microbiome or urobiome [1,2].

Consequently, the contemporary definition of a UTI is not merely a simple invasion, but a state of microbial dysbiosis. This dysbiosis occurs when the normal balance between protective commensal flora and transient pathobionts is disrupted. This disruption enables the overgrowth of uropathogens, predominantly UPEC, which can breach the urothelial barrier, trigger robust inflammatory cascades via Toll-like receptor (TLR) signalling, and result in clinical symptomatology [3,4].

Furthermore, pathogenesis is no longer viewed only as an extracellular process. Recent molecular evidence demonstrates that uropathogens invade the bladder urothelium and establish two distinct intracellular populations: replicative intracellular bacterial communities (IBCs) within the superficial umbrella cells, and quiescent intracellular reservoirs within the deeper, less differentiated epithelium. These intracellular niches protect bacteria from immune surveillance and systemic antibiotics, and together they redefine our understanding of chronicity and recurrence in UTIs [4,5].

## 2. Etiological Agents: The Microbial Landscape of Uropathogenicity

The etiology of urinary tract infections is defined by a group of microorganisms capable of passing the host defences of the urinary tract. While a broad spectrum of bacteria, fungi, and viruses can occasionally invade this niche, the clinical burden is dominated by a select group of uropathogens possessing specific molecular characteristics for adhesion, nutrient acquisition, and immune evasion [6,7].

The hierarchy of uropathogens is consistently led by UPEC, which represents 70–80% of uncomplicated cases and a large proportion of complicated infections [1,7]. UPEC’s prevalence is attributed to its vast repertoire of virulence factors (VFs), including siderophores for iron sequestration and an array of chaperone-usher fimbriae [8,9].

Beyond UPEC, other primary agents contribute significantly to the clinical spectrum.

*Klebsiella pneumoniae*, which is a frequent cause of both community- and hospital-acquired UTIs, is well known for its thick polysaccharide capsule that resists phagocytosis and for carrying multidrug-resistant (MDR) plasmids [10,11].

*Proteus mirabilis* is distinguished by its characteristic swarming motility and urease production. This enzyme hydrolyzes urea into ammonia, raising urinary pH and precipitating calcium and magnesium phosphate stones, which serve as protected sites for bacterial persistence; in a murine model of ascending infection, urease contributed directly to urolithiasis and pyelonephritis [12,13]. *Enterococcus faecalis* is a Gram-positive commensal of the gastrointestinal tract that has emerged as a major uropathogen, particularly in catheterized patients, owing to its resistance to many antibiotics and its capacity to form a robust biofilm [14,15]. Finally, *Staphylococcus saprophyticus* is a coagulase-negative Staphylococcus that exhibits a specific tropism for the uroepithelium, primarily causing uncomplicated cystitis in young, sexually active women [16,17].

Community-Acquired vs. Nosocomial Pathogens

The etiological distribution of uropathogens can shift between community-acquired and healthcare-associated UTIs, with nosocomial infections reflecting a greater microbial diversity and significantly higher antimicrobial resistance rates [18]. These infections are generally caused by *Pseudomonas aeruginosa*, *Enterobacter species*, and yeast such as *Candida albicans*, often facilitated by the presence of urinary catheters [19,20]. Recent studies have explained the mechanisms by which catheterization facilitates infection with *Candida albicans*. Catheter-induced inflammation promotes the deposition of fibrinogen on the bladder mucosa, which the organism subsequently uses as a scaffold for biofilm formation. The process depends on the transcription factor Efg1 and the adhesin Als1, both of which mediate fibrinogen-associated biofilm development. Furthermore, successful infection in the catheterized bladder requires both morphological transition and effective epithelial attachment, which reflects the multifactorial nature of pathogenicity [21].

The ESKAPE Group and Global Resistance Trends

A critical “State of the Art” concern in 2026 is the rising prevalence of the ESKAPE pathogens, which include *Enterococcus faecium*, *Staphylococcus aureus*, *Klebsiella pneumoniae*, *Acinetobacter baumannii*, *Pseudomonas aeruginosa*, and *Enterobacter species*. They represent the primary drivers of the “post-antibiotic era” in urology, with therapeutic challenges due to their multidrug resistance, extensive drug resistance (XDR), and in some cases pandrug resistance (PDR) [22]. Resistance among ESKAPE pathogens is driven by multiple mechanisms that together limit antimicrobial efficacy. A principal strategy involves drug inactivation through the production of β-lactamases, including extended-spectrum β-lactamases (ESBLs), AmpC enzymes, and carbapenemases such as *Klebsiella pneumoniae* carbapenemase (KPC), OXA, New Delhi metallo-β-lactamase (NDM), Verona integron-encoded metallo-beta-lactamase (VIM), and Active on Imipenem (IMP). In addition, target modification plays a crucial role, for example, by altering penicillin-binding proteins in methicillin-resistant *Staphylococcus aureu* (MRSA), ribosomal modifications conferring aminoglycoside resistance, and mutations in DNA gyrase or topoisomerase IV associated with fluoroquinolone resistance. Reduced membrane permeability, particularly via porin loss or mutation in Gram-negative organisms, restricts antibiotic entry, while upregulation of efflux pumps actively removes antimicrobial agents across multiple pathogen groups. Finally, the creation of biofilm contributes significantly to resistance because it forms a protected microenvironment that impairs antibiotic penetration and promotes bacterial persistence [23,24].

## 3. Molecular Determinants of Uropathogenicity

The capacity of an organism to establish infection in the urinary tract depends less on its abundance in the fecal reservoir than on whether it carries the molecular equipment to attach to the urothelium, acquire iron under severe restriction, resist expulsion, and survive the innate immune response. The following sections deal with those functions in turn, using UPEC as the reference organism and noting where other uropathogens converge on the same solutions by different routes.

### 3.1. Adhesion: The Committed Step

Attachment is the point at which colonization becomes an infection. Urine flow removes non-adherent bacteria efficiently, so an organism that cannot bind is cleared regardless of how many virulence genes it carries.

Type 1 pili mediate the initial event. These chaperone-usher fimbriae terminate in the two-domain adhesin FimH, whose lectin domain binds mannosylated residues on uroplakin Ia, a major structural protein of the apical membrane of superficial umbrella cells [25]. The binding behaves as a catch bond: tensile force applied across the adhesin separates the lectin domain from the pilin domain and shifts FimH into a high-affinity conformation, so that shear stress strengthens rather than weakens attachment [26]. This is a counterintuitive but clinically important point, since it explains why increased urine flow does not remove adherent bacteria and why advice to increase fluid intake, whatever its other merits, does not mechanically remove organisms that are already bound. Allelic variation in fimH further separates uropathogenic from commensal lineages; point mutations in the lectin domain that increase affinity for monomannose structures are enriched among urinary isolates and appear to be positively selected during infection [27].

P fimbriae govern the second, more consequential step. The tip adhesin PapG binds the Galα(1–4)Gal moiety of globoseries glycolipids, which are expressed at high density on renal epithelium and on erythrocytes of the P blood group [28]. Three allelic classes exist, and they are not interchangeable: class II PapG, which recognizes globotetraosylceramide, is strongly associated with human pyelonephritis, whereas class III, which binds globopentaosylceramide, is more often recovered from cystitis and from pediatric isolates [29]. This distinction is the mechanistic reason that the ascent from bladder to kidney is a property of particular strains rather than a stochastic event, and it accounts for much of the clinical behaviour attributed below to phylogroup B2, in which pap operons are concentrated.

Other uropathogens solve the same problem with unrelated structures. *Enterococcus faecalis* expresses endocarditis- and biofilm-associated pili whose adhesin EbpA binds fibrinogen deposited on the catheter surface following mechanical irritation of the bladder mucosa [30]. The parallel with the Efg1- and Als1-dependent binding of *Candida albicans* to catheter-associated fibrinogen, described in Section 2, is close enough to suggest that host fibrinogen deposition, rather than the device material itself, is the shared substrate for catheter-associated infection across bacterial and fungal uropathogens.

### 3.2. Toxin-Mediated Injury and Cytoskeletal Subversion

α-Haemolysin (HlyA), an RTX-family pore-forming toxin, exerts distinct effects according to concentration. At lytic concentrations it destroys urothelial and immune cells outright. At sublytic concentrations it acts as a signalling toxin, inducing oscillatory calcium fluxes in renal epithelial cells that alter cytokine production [31], and promoting the degradation of host cytoskeletal and signalling proteins through activation of endogenous proteases, with consequent inhibition of pro-survival signalling and exfoliation of the superficial cell layer [32]. Exfoliation is double-edged: it removes colonized cells, but it also exposes the underlying transitional epithelium, which is both more permissive to invasion and the compartment in which persistent reservoirs are later established.

Cytotoxic necrotizing factor 1 (CNF1) acts by deamidating a single glutamine residue in Rho-family GTPases, locking RhoA, Rac1 and Cdc42 in their active states [33]. The resulting actin reorganization and membrane ruffling facilitate bacterial internalization, so the toxin functions less as a cytotoxin than as an entry factor.

### 3.3. Iron Acquisition and the Siderophore–Lipocalin Conflict

Free iron in urine is negligible, and competition for it has produced one of the clearest examples of molecular escalation in this system. UPEC secretes enterobactin, a catecholate siderophore of very high affinity for ferric iron. The host counters with lipocalin-2 (neutrophil gelatinase-associated lipocalin), which binds ferric enterobactin directly and denies the bacterium its cargo [34]. Uropathogenic strains in turn carry the iroA locus, whose products C-glucosylate enterobactin to yield salmochelin; the glycosylation is sterically incompatible with the lipocalin-2 binding pocket, and the modified siderophore is recovered through its own receptor, IroN [35]. Two further systems, aerobactin (receptor IutA) and yersiniabactin (receptor FyuA), broaden the repertoire, with the latter having the additional property of chelating copper and thereby mitigating copper-mediated killing within the phagosome [36].

### 3.4. Innate Defence of the Urothelium

Host resistance is not passive. Uromodulin, the most abundant protein in normal urine, carries high-mannose glycans that act as a soluble decoy for FimH, aggregating type 1 piliated bacteria and promoting their removal; mice deficient in uromodulin show increased bacterial burdens after transurethral challenge [37]. Toll-like receptor 4 recognizes lipopolysaccharide at the apical urothelial surface and initiates the cytokine response, and it additionally drives a cAMP-dependent expulsion of bacteria that have already been internalized within fusiform vesicles, returning them to the lumen before they reach the cytosol [38]. Antimicrobial peptides constitute a third layer: cathelicidin LL-37 is produced constitutively and upregulated on bacterial contact, with cathelicidin-deficient animals showing markedly greater susceptibility [39], while RNase 7 and human β-defensin-1 contribute additional constitutive activity [40,41].

These defences also explain the pathogen’s counter-strategy. Escape into the cytosol, discussed next, removes the organism from the compartment in which TLR4-mediated expulsion and secreted antimicrobial peptides operate.

### 3.5. Intracellular Bacterial Communities and the Persistent Reservoir

Following FimH-mediated attachment, UPEC is internalized into superficial umbrella cells within fusiform vesicles. Escape into the cytosol depends on disruption of the vesicle membrane by the outer membrane phospholipase PldA [42]. Once cytosolic, the organisms replicate rapidly and coalesce into tightly packed, biofilm-like intracellular bacterial communities, in which a single infected cell may harbour on the order of 10^4^ organisms [43]. The matrix confers substantial tolerance to antibiotics that are bactericidal against the same strain in planktonic culture, which is one reason microbiological cure and clinical cure diverge in recurrent disease.

Dispersal from a mature community is accompanied by a striking morphological change: a subpopulation adopts a filamentous form, tens of micrometres in length, which resists neutrophil phagocytosis and permits spread to neighbouring cells, where the cycle repeats [44,45]. A further subset does not disperse but establishes quiescent intracellular reservoirs within the underlying transitional epithelium, where organisms persist in a non-replicating state, sheltered from both antibiotic exposure and immune surveillance, and from which reactivation can occur weeks to months later [46].

This model provides a coherent mechanistic account of recurrence with a genetically identical strain after documented microbiological clearance and reframes such episodes as reactivation rather than reinfection. It should be said plainly, however, that most of the evidence derives from murine models. Human data are more limited, though the recovery of viable organisms and the lymphocytic infiltrates demonstrated in bladder biopsies from women with recurrent infection are consistent with the same process [47,48].

### 3.6. Persistence Mechanisms in Non-Escherichia coli Uropathogens

*Proteus mirabilis* depends on urease, which is transcriptionally controlled by the urea-responsive regulator UreR [12]. Hydrolysis of urea raises urinary pH and precipitates struvite and carbonate apatite, producing stones that enclose viable organisms in a compartment inaccessible to antibiotics; stone clearance, not antimicrobial therapy, is therefore the determinant of cure. The organism additionally undergoes swarmer cell differentiation, elongating and hyperflagellating on contact with surfaces, which supports migration along catheters [49].

In *Klebsiella pneumoniae*, the polysaccharide capsule impedes complement deposition and phagocytic uptake, and hypermucoviscous variants show correspondingly greater resistance to serum killing [50].

### 3.7. Molecular Basis of Resistance and Biofilm Tolerance

The resistance mechanisms summarized in Section 2 warrant specification at the molecular level. Efflux in Enterobacterales is dominated by the tripartite AcrAB–TolC system, whose overexpression following derepression of local and global regulators reduces susceptibility across structurally unrelated agents simultaneously [51]. Reduced permeability is most consequential in *K. pneumoniae*, where loss or mutation of the porins OmpK35 and OmpK36 combined with ESBL or AmpC production yields carbapenem resistance without any carbapenemase [52], a phenotype that is readily missed if resistance is inferred from genotype alone.

Biofilm tolerance rests on a defined extracellular matrix. In UPEC this comprises curli amyloid fibres, encoded by the csg operons, together with cellulose, the two forming a composite that limits antibiotic penetration and maintains a metabolically heterogeneous population in which slow-growing subpopulations survive exposure that eliminates their actively dividing neighbours [53]. Tolerance of this kind is phenotypic rather than genetic, and it will not be detected by standard susceptibility testing, a discrepancy that goes some way towards explaining treatment failure in catheter-associated infection despite an apparently susceptible isolate.

Having set out the individual determinants of uropathogenicity, we now consider how they are distributed across the phylogenetic structure of the species, since it is the combination of these factors in particular lineages, rather than any single gene, that predicts clinical behaviour.

## 4. Molecular Classification

The molecular classification of *Escherichia coli* into phylogenetic groups provides important information for understanding the pathogenic potential of strains involved in urinary tract infections, as described by Clermont et al. These phylogroups reflect evolutionary lineages that differ considerably in their genetic content, virulence capacity, and clinical impact. Importantly, they can be arranged along a continuum ranging from largely commensal and low-virulence strains to highly adapted uropathogenic lineages [54,55,56].

At the least virulent end of the spectrum are phylogroups A and B1, which are traditionally considered commensal strains of the intestinal microbiota. These groups generally lack the complex virulence gene repertoires required to establish infection in an otherwise healthy urinary tract. However, they can become opportunistic pathogens under specific conditions, particularly in patients with compromised host defences or structural abnormalities of the urinary tract. Their involvement in UTIs is often associated with the gathering of mobile genetic elements, such as plasmids carrying multidrug-resistant genes. In this context, their pathogenicity is driven less by intrinsic virulence and more by their ability to survive antibiotic pressure and persist in vulnerable hosts [54,55,56].

Closely related to these groups is phylogroup C, which shares many genetic features with B1 and is similarly considered to have low baseline virulence, as shown by a Mash-based analysis of 10,667 *E. coli* genomes from GenBank. Strains in this group are not typically primary uropathogens but may contribute to infections in complicated clinical scenarios, such as catheter-associated UTIs or infections in immunocompromised individuals. Like A and B1, their clinical relevance is increasing due to their capacity to acquire resistance determinants, enabling persistence and treatment failure despite limited inherent pathogenicity [55,57,58].

Moving toward intermediate virulence, phylogroup E represents a less well-characterized lineage that exhibits features of both commensal and pathogenic strains. Although not as extensively studied, some E strains have been found to carry virulence-associated genes and to participate in extraintestinal infections, including UTIs. Their pathogenic potential appears variable and context-dependent, suggesting that they may act as opportunistic pathogens with the possibility to evolve toward increased virulence [55,59,60,61].

A more recently recognized group, phylogroup G, remains incompletely understood but is gaining attention in molecular epidemiology studies. Early evidence suggests that some strains within this group possess traits associated with extraintestinal adaptation, including virulence and survival mechanisms relevant to urinary tract colonization. Particularly, STc117 lineages have multiple virulence-associated determinants characteristic of extraintestinal pathogenic *E. coli*. (ExPEC) and have demonstrated pathogenicity in murine sepsis models. In addition, many ST117 isolates exhibit multidrug resistance, frequently associated with CTX-M-type extended-spectrum β-lactamases, although resistance profiles may vary across isolates [59,62].

At the higher end of the virulence spectrum lies phylogroup D, which represents a well-established source of extraintestinal pathogenic *E. coli* (ExPEC), accounting for 8.6–31.3% of uropathogenic isolates across different geographic regions [63]. Strains in this group have a more complex set of virulence factors compared to commensal phylogroups, including adhesins, toxins, and iron acquisition systems. In many studies comparing UTI isolates to fecal flora, phylogroup D (along with B2) showed significantly higher representation among clinical isolates, while commensal strains predominantly belonged to phylogroup A [64]. These characteristics help with the colonization of the urinary tract, evasion of host immune responses, and tissue damage. While generally less virulent than B2 strains, phylogroup D organisms are nonetheless significant contributors to both uncomplicated and complicated UTIs. As seen in one large study, B2 strains averaged 10.1 virulence genes per isolate, while phylogroup D had lower average scores [59,64].

Closely related to the most virulent lineage is phylogroup F, which has emerged as an important and concerning group in recent years. Phylogroup F shares many characteristics with B2 strains, including the presence of multiple virulence-associated genes, but is also frequently associated with multidrug resistance [59]. This combination of high virulence and resistance makes F strains particularly problematic in clinical settings, especially in recurrent and treatment-resistant infections. Urovirulence is most often observed in B2/F/D group strains and represents a multigenic process involving numerous combinations of genes and specific alleles with epistatic interactions [65]. As such, phylogroup F represents a hybrid that combines both intrinsic virulence and adaptive resistance mechanisms [55,59].

**Figure 1:**
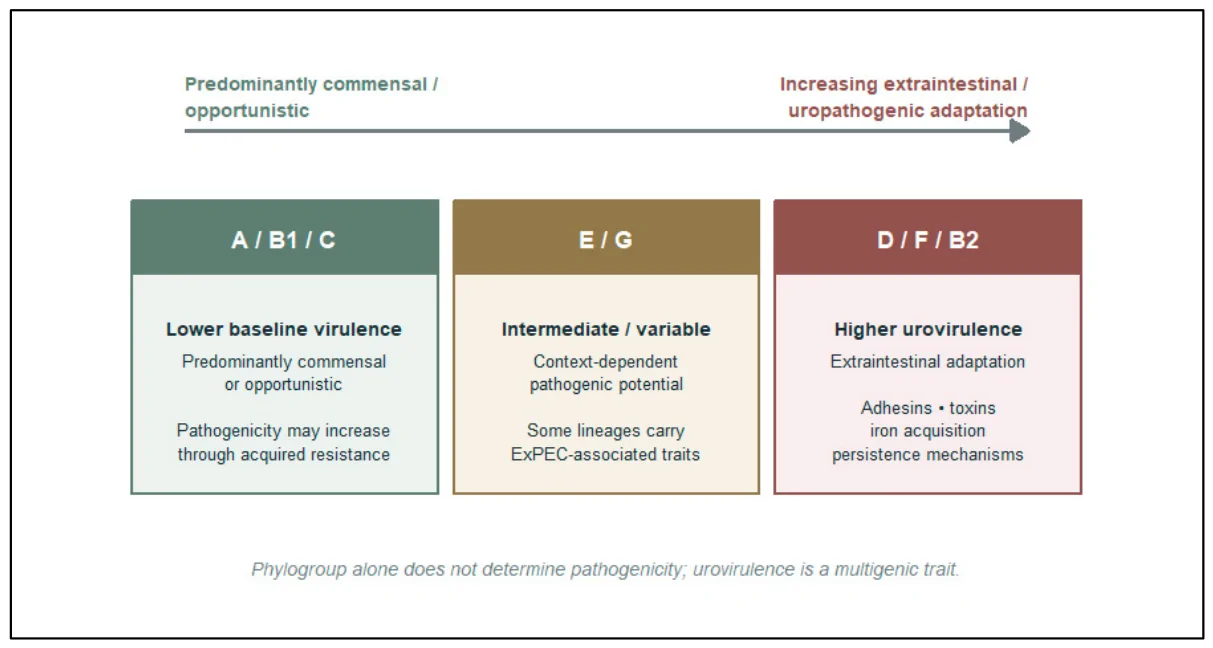
Molecular classification of *Escherichia coli* phylogroups according to uropathogenic potential.

Phylogroup B2 constitutes the principal reservoir of UPEC and carries the highest average virulence factor gene burden (10.1 genes/isolate) among all phylogroups, as seen in Figure 1 [59]. It consistently predominates across diverse geographic regions and clinical settings, accounting for 64.8% of UPEC isolates in Taiwan, 77.7% of pyelonephritis isolates in Korea, 46.1% of complicated UTI isolates in Malaysia, and 30% of UTI isolates in Ethiopia [59,66,67,68]. B2 strains are highly specialized for extraintestinal infection and are characterized by a dense accumulation of pathogenicity islands encoding a wide array of virulence factors. These include adhesins such as P fimbriae, toxins like α-hemolysin, and sophisticated iron acquisition systems that allow survival in the nutrient-limited environment of the urinary tract [59,64,68]. In addition, B2 strains are capable of developing intracellular bacterial communities and biofilms, contributing to persistence and recurrence of infection. Their evolutionary adaptation makes them the most efficient and aggressive UTI pathogens [69].

## 5. Clinical Classification

The current clinical classification of UTIs has evolved significantly, with both the European Association of Urology (EAU) and the Infectious Diseases Society of America (IDSA) 2025 guidelines now focusing on whether infection is localized, that is, uncomplicated, versus systemic, also known as complicated, rather than traditional host-based risk factors alone [70,71].

The 2025 IDSA guidelines represent a paradigm shift in UTI classification:

Uncomplicated UTI (uUTI): An infection that appears confined to the bladder on initial evaluation, without systemic signs of illness. Importantly, patients with underlying urologic abnormalities (benign prostatic hypertrophy, cystocele), diabetes, or immunocompromise are not automatically classified as complicated if they appear to have bladder-confined infection and are not systemically ill. By this classification, both men and women can have uncomplicated UTI, though antibiotic choice and duration may differ [71].

Complicated UTI (cUTI): An infection with systemic signs of illness that can include fever and bacteremia, evidence of renal parenchymal involvement with costovertebral angle tenderness, prostatic abscess, or subsequent discovery of upper urinary tract obstruction [71].

**Figure 2:**
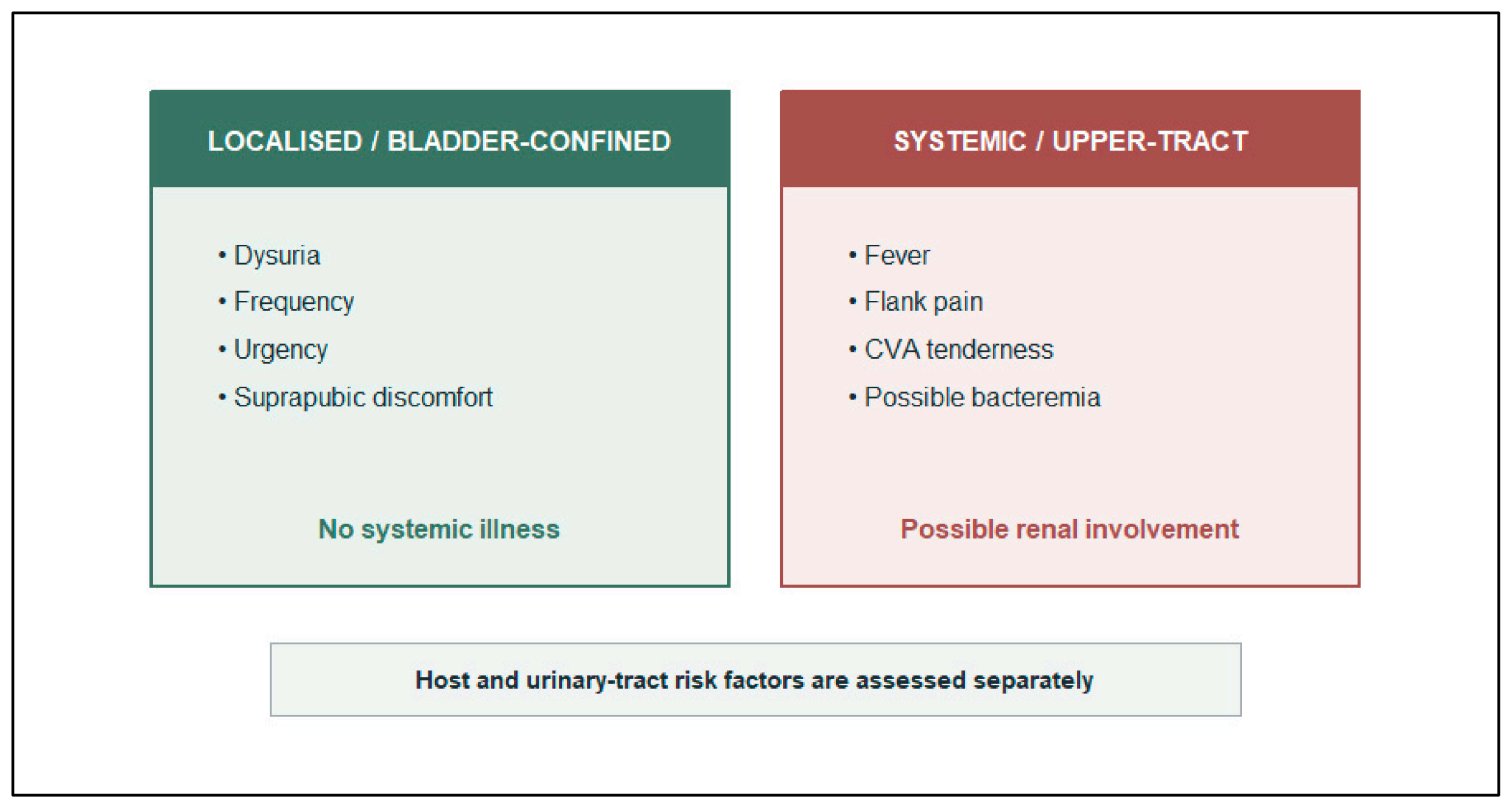
Clinical classification of urinary tract infections.

The EAU Urological Infections Guidelines Panel has proposed aligning classifications as localized versus systemic, with risk factors for treatment failure considered separately for both categories [70,71]. The differences between the two categories are summarized in Figure 2: patients with complicated UTI characteristically have pain higher in the urinary tract, together with systemic features such as fever.

Lower urinary tract infections, particularly acute uncomplicated cystitis, are described by a well-defined constellation of localized urinary symptoms that reflects the inflammation of the bladder mucosa. Clinically, patients usually present with dysuria, described as a burning sensation during urination, accompanied by increased urinary frequency and urgency due to the irritation of the bladder. Suprapubic discomfort or pain is also commonly reported, reflecting underlying bladder inflammation. In some cases, hematuria may be observed; it can range from microscopic to gross, reflecting mucosal irritation and inflammation [72]. New-onset dysuria and frequency represent the most specific symptom pattern for UTI diagnosis [72].

An upper urinary tract infection, or pyelonephritis, involves the renal parenchyma and pelvis.

Acute pyelonephritis is a severe UTI involving the renal parenchyma and pelvis, with the potential to cause sepsis, septic shock, and death. The estimated annual incidence is 459,000 to 1,138,000 cases in the United States and 10.5 to 25.9 million cases globally [73].

In pregnant individuals, the American College of Obstetricians and Gynecologists (ACOG) recommends that pyelonephritis should be suspected in the presence of fever ≥ 38.0 °C and urine studies suggesting UTI, with additional symptoms of upper genitourinary tract infection such as flank pain or costovertebral angle tenderness, which support the diagnosis [74]. In patients presenting with flank pain or costovertebral angle tenderness, with or without fever, a positive urinalysis demonstrating pyuria, bacteriuria, or both supports a presumptive diagnosis of acute pyelonephritis, irrespective of the presence of lower urinary tract symptoms [73]. The clinical presentation is notably heterogeneous. The classic manifestation involves the acute onset of systemic inflammatory features, including fever, chills, and malaise, accompanied by lower urinary tract symptoms such as urinary frequency, urgency, and dysuria. However, atypical presentations are not uncommon; up to 20% of patients may lack bladder symptoms, and a subset may present without fever. The severity of illness spans a broad clinical spectrum, ranging from mild flank discomfort with low-grade fever to severe systemic involvement, including sepsis and septic shock [73].

The 2025 guidelines explicitly state that patients with underlying urologic abnormalities, which could include benign prostatic hypertrophy and/or cystocele, diabetes, or immunocompromise, are not automatically classified as having complicated UTI [71]. Where infection is localized to the bladder and the patient is not systemically ill, the episode is considered uncomplicated. The new definition moves us away from the traditional definition, which classified UTI as complicated based on the presence of any host factor that might increase risk of treatment failure [75].

An international consensus statement has identified key risk factors associated with the development of urinary tract infections, as well as with increased disease severity and a higher likelihood of treatment failure. Foremost among these is the presence of a urethral catheter, which represents the most significant predisposing factor. Additional important contributors include structural abnormalities of the urinary tract that impair effective urinary drainage, as well as the presence of urolithiasis, both of which facilitate bacterial persistence and complicate clinical management [76,77].

Structural and functional abnormalities of the urinary tract undermine intrinsic host defence mechanisms through multiple pathophysiological processes [77]. A central factor is the impairment of mechanical clearance, as continuous urine flow constitutes a critical defence against microbial colonization. Conditions associated with obstruction or with urinary stasis facilitate bacterial persistence and proliferation within the urinary tract [77]. Experimental evidence further supports this mechanism, demonstrating that transurethrally introduced bacteria are rapidly cleared from a physiologically normal bladder, whereas the presence of urethral obstruction predisposes to ascending infection, resulting in cystitis, pyelonephritis, and, in severe cases, bacteremia [77,78,79].

Recurrent UTI (rUTI) is defined as ≥2 UTIs within 6 months or ≥3 UTIs within 12 months, with at least one episode being culture-positive to confirm infectious etiology. Over 50% of adult women experience at least one UTI in their lifetime, and nearly one-quarter will experience recurrent UTI [71].

In a large cohort study of 374,171 women with index uncomplicated UTI, 14.5% developed recurrent UTI [80]. Risk factors associated with recurrent urinary tract infections encompass a range of demographic, clinical, and microbiological variables. Age represents a significant determinant, with both younger women between 18 and 27 years and elderly individuals over ≥78 years demonstrating an increased susceptibility to recurrence [80,81]. Microbiological factors, including a positive urine culture at the time of the index UTI and infection with antibiotic-resistant organisms, are associated with a higher rate of recurrences. Premenopausal women, sexual intercourse ≥3 times weekly, spermicide use, new or multiple sex partners, and a UTI before age 15 are all known risk factors. In postmenopausal women, risk is primarily increased by the lower estrogen levels [81].

**Figure 3:**
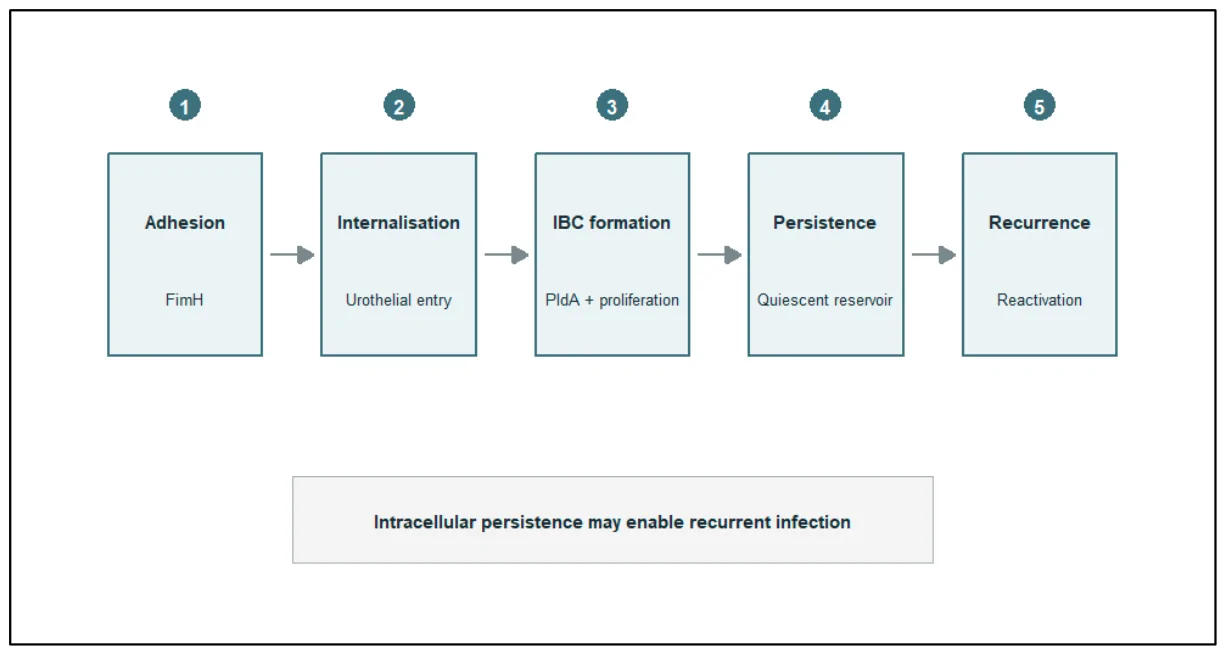
Intracellular persistence as a mechanism of recurrent UPEC infections.

As described in Section 3.5, the intracellular bacterial communities and quiescent intracellular reservoirs that underlie recurrence form in distinct urothelial compartments; the clinical consequences of this persistence are considered here (Figure 3).

Direct clinical and histopathological evidence supports the persistence of bacteria within the human bladder wall in patients with recurrent urinary tract infections [47]. Studies have demonstrated that viable bacteria can be isolated from bladder tissue of postmenopausal women with rUTIs, often showing a diverse range of bacterial species rather than a single dominant pathogen [48]. Histopathological analyses further reveal pronounced inflammatory and structural changes, including tissue edema, disruption of normal urothelial architecture, and infiltration by immune cells, particularly lymphocytes and plasma B cells, which indicates a chronic inflammatory response [47]. At the microbiological level, strains associated with recurrent infections have shown a significantly greater intracellular bacterial burden compared with sporadic infections, suggesting enhanced capacity for intracellular survival and persistence [47,82].

Another problem that a clinician is confronted with is distinguishing asymptomatic bacteriuria (ASB) from symptomatic UTI. The clinical spectrum ranges from ASB to symptomatic UTI to urosepsis. In older adults, the critical distinction lies in the presence or absence of localizing urinary symptoms [83]. In older adults, the diagnosis of symptomatic UTI is primarily clinical and requires the exclusion of alternative causes. Patients presenting with at least two of the following features meet the diagnostic criteria: fever, worsening urinary urgency or frequency, acute dysuria, suprapubic tenderness, or costovertebral angle pain or tenderness [71]. Microbiological confirmation is carried out using a positive urine culture demonstrating ≥10^5^ CFU/mL with no more than two uropathogens, in combination with the presence of pyuria, thereby supporting the diagnosis [71,83].

The prevalence of ASB can vary considerably across different populations, reflecting underlying host factors and exposure risks. It is quite uncommon in healthy premenopausal women, with reported rates ranging from 1 to 5%, but rises in pregnant women from approximately 2 to 10% [84]. A significant rise is observed in older women (>65 years), among whom prevalence exceeds 15%. Even higher rates are documented in institutionalized populations, with up to 50% of residents in long-term care facilities affected. Notably, in patients who have urinary catheters, bacteriuria becomes common with prolonged catheterization, proving the strong association between device use and microbial colonization [84,85].

**Figure 4:**
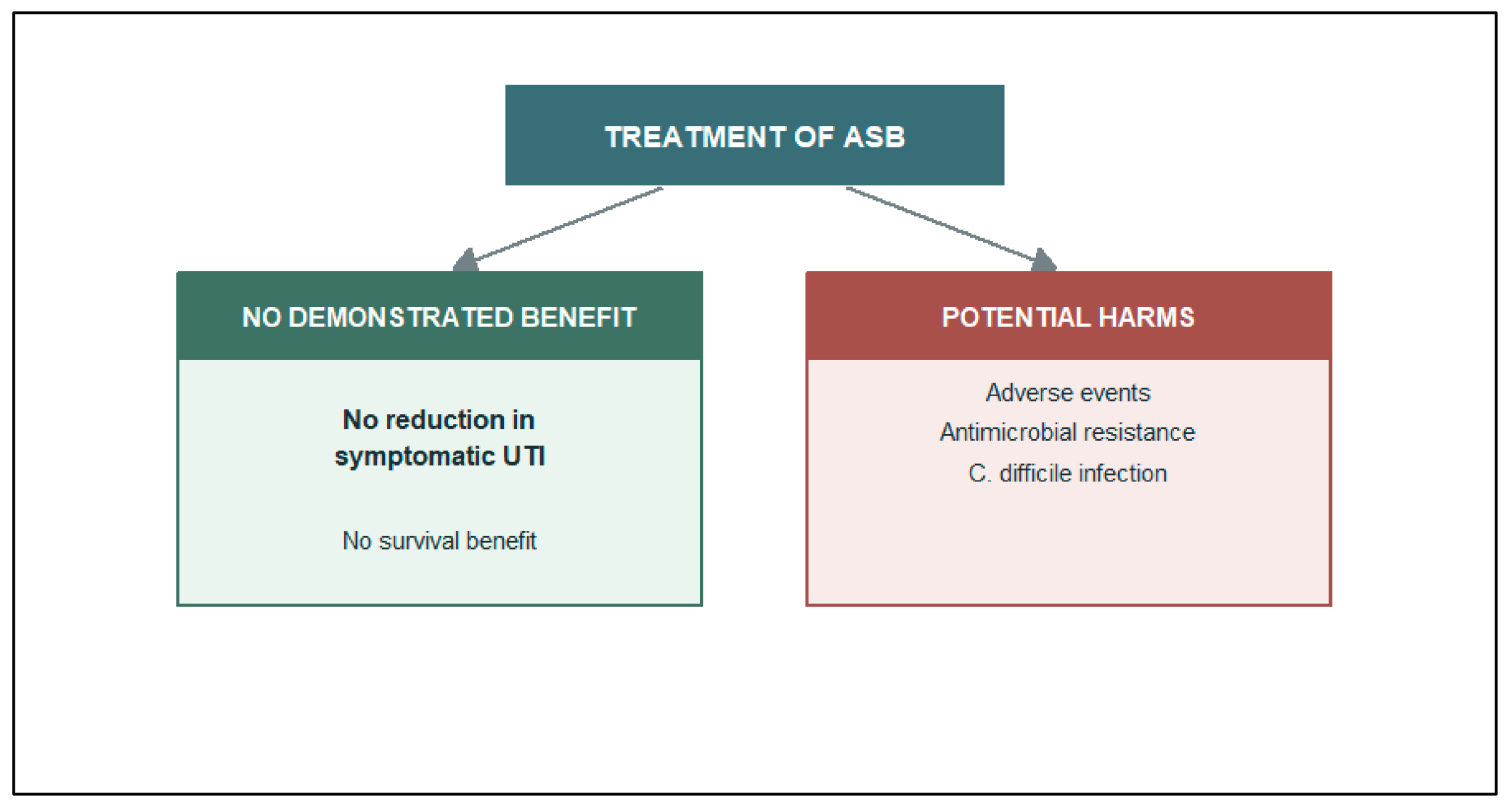
Clinical consequences of antimicrobial treatment for asymptomatic bacteriuria.

Distinguishing ASB from symptomatic UTI matters clinically, because treating ASB is harmful in most populations. Multiple studies demonstrate that treating ASB in low-risk populations provides no benefit and causes harm [84,86]. Evidence from prospective randomized trials indicates no reduction in the incidence of subsequent symptomatic urinary tract infections between treated and untreated individuals, as also shown in Figure 4. Similarly, no mortality benefit has been observed; a prospective study involving 358 older adults reported no significant effect on survival. Moreover, treatment is associated with an increased risk of adverse events, including dermatologic reactions, candidiasis, and gastrointestinal disturbances such as diarrhea [86]. Importantly, inappropriate antimicrobial use contributes to the development of antimicrobial resistance and increases the risk of Clostridioides difficile infection [87].

## 6. Diagnostic Methods in Urinary Tract Infections

The diagnosis of UTI integrates clinical presentation, urinalysis (dipstick and microscopy), and urine culture, while imaging is reserved for specific indications. The diagnostic approach varies by patient population and clinical context.

From the clinical point of view, the probability of cystitis exceeds 90% in women presenting with dysuria and frequency without vaginal discharge or irritation. Dysuria is also common with urethritis or vaginitis, but cystitis is more likely when symptoms are sudden or severe in onset and vaginal irritation/discharge are absent [72].

Laboratory testing is an essential part of diagnostic testing. Dipstick testing detects leukocyte esterase and nitrites [88]. Nitrite testing is unreliable, however, as not all uropathogens such as *Enterococcus*, *Pseudomonas*, and *Staphylococcus saprophyticus* produce nitrites [88]. Additionally, pyuria may be absent in early infection or in immunocompromised states, such as neutropenia. False-positive pyuria is also common in older adults, particularly in the presence of non-infectious lower urinary tract symptoms [88]. In older patients, dipstick testing demonstrates high sensitivity (~90%) but poor specificity (~56%) for bacteriuria, limiting its utility in confirming UTI [89].

The next step is urine microscopy, which evaluates for pyuria (>5 WBC/high-power field) and bacteriuria. In young children and infants, the combination of dipstick and microscopy has been reported to achieve a sensitivity of 99.8% with a specificity of 70% [90]; comparable combined estimates in adults are lower and less consistent. Pyuria alone is likely the best marker in terms of negative predictive value, though methods for detection have not been standardized [91].

The gold standard of today remains the urine culture for the diagnosis of urinary tract infection and for guiding the selection of targeted antimicrobial therapy through susceptibility testing. Diagnostic thresholds vary according to clinical context and specimen collection methods. Traditionally, a threshold of ≥10^5^ CFU/mL has been used for symptomatic patients; however, lower cutoffs (10^4^–10^3^ CFU/mL) are increasingly accepted in many laboratories, particularly in specific populations or with optimized collection techniques [91]. Importantly, in symptomatic individuals, the presence of as few as 10^2^ CFU/mL of a single uropathogen may be clinically significant and indicative of true infection [92].

In the UTI diagnostic routine, imaging is not indicated for uncomplicated UTI or uncomplicated pyelonephritis. It comes between ultrasound and computer tomography, which is used in complicated situations. CT findings in pyelonephritis include renal swelling, perinephric fluid, hydronephrosis, and focal areas of decreased enhancement. CT has a detection rate of 84% compared to 40% for ultrasound in acute pyelonephritis [93].

## 7. Therapeutic Management of Uncomplicated and Complicated Urinary Tract Infections

First-line empiric therapy for uncomplicated cystitis includes nitrofurantoin for 5 days, trimethoprim/sulfamethoxazole for 3 days, or fosfomycin as a single dose, all with comparable efficacy [94,95]. The IDSA/ESCMID international clinical practice guidelines also recommend pivmecillinam, at 400 mg twice daily for 3–7 days, as a first-line agent where available [96]. Men presenting with UTI symptoms should always undergo urine culture with susceptibility testing, and prostatic involvement should be considered in every case, since it changes both agent selection and treatment duration. Where infection is reliably confined to the bladder and prostatitis has been excluded, trimethoprim/sulfamethoxazole or trimethoprim are appropriate first-line options. Nitrofurantoin may be used only in this setting because, as it does not achieve therapeutic concentrations in prostatic tissue, it is unsuitable whenever prostatic involvement is suspected or has not been excluded. When prostatitis is present, an agent with adequate prostatic penetration, such as a fluoroquinolone or trimethoprim/sulfamethoxazole, is required, and a longer course is needed [92].

But in the case of a complicated UTI, the situation is completely different. The Infectious Diseases Society of America recommends a structured four-step approach to guide empiric antibiotic selection. This includes assessment of illness severity; individual risk factors for antimicrobial resistance, such as recent antibiotic exposure; whether there was any prior infection with resistant organisms; and whether the patient has any drug allergies or potential drug interactions. Renal function should be checked, and local antimicrobial therapy should be incorporated using the antibiogram [71]. A distinction must also be drawn between patients with and without sepsis. Thus, for complicated urinary tract infections without sepsis, the standard therapy typically includes broad-spectrum agents such as third- or fourth-generation cephalosporins, piperacillin–tazobactam, or fluoroquinolones [71]. Carbapenems and newer antimicrobial agents including ceftolozane–tazobactam, ceftazidime–avibactam, cefiderocol, and plazomicin should be reserved only for cases involving suspected or confirmed multidrug-resistant organisms [97]. In contrast, for cUTIs presenting with sepsis, empiric treatment must ensure adequate and timely coverage due to the increased risk of adverse outcomes. Recommended options include third- or fourth-generation cephalosporins, piperacillin–tazobactam, fluoroquinolones, and carbapenems, with the latter considered first-line in this setting given the critical importance of avoiding inadequate initial therapy [71,98].

## 8. Emerging Therapeutic Trends in Urinary Tract Infections: Molecular and Clinical Perspectives

Emerging therapeutic trends in UTI management reflect a shift toward microbiome-centred approaches, novel antimicrobial agents with unique mechanisms, non-antibiotic prevention strategies, and immunoprophylaxis, caused by rising antimicrobial resistance and the need for antimicrobial stewardship [99].

Research into novel antimicrobial agents has produced encouraging results. Gepotidacin is a first-in-class triazaacenaphthylene antibiotic that is FDA-approved for uncomplicated UTI in females ≥ 12 years. Its mechanism involves dual inhibition of DNA gyrase and topoisomerase IV through a distinct binding site, requiring mutations in both enzymes for resistance to develop—a feature that may preserve long-term effectiveness [100,101]. Pivmecillinam, FDA-approved for uncomplicated UTI in women ≥18 years, has been used extensively in Scandinavia for decades with minimal resistance development and low collateral damage to the microbiome. It selectively binds penicillin-binding protein 2 and is effective against most Enterobacterales [96,100]. Third, cefiderocol is a siderophore cephalosporin approved for complicated UTI and hospital-acquired pneumonia; it exploits bacterial iron transport systems to penetrate the outer membrane of Gram-negative bacteria, including carbapenem-resistant organisms [99].

Non-antibiotic prevention strategies aim at enhancing the protective urogenital microbiome rather than solely eradicating pathogens. For example, methenamine is converted to formaldehyde in acidic urine, providing bacteriostatic activity. In a multicentre, open-label, randomized non-inferiority trial conducted in secondary care, methenamine hippurate was non-inferior to daily low-dose antibiotic prophylaxis in adult women with recurrent UTI [102]. A subsequent triple-blind, placebo-controlled trial in women aged 70 years and older recruited from general practice reported a reduction of approximately 25% in antibiotic-treated UTI episodes during treatment, although the incidence of UTI rose after discontinuation [103]. D-mannose is a monosaccharide long thought to inhibit bacterial adherence to uroepithelial cells by binding the FimH adhesin, and it has been widely recommended on that mechanistic basis. However, a recent randomized placebo-controlled trial found no significant benefit of D-mannose over placebo for preventing recurrent UTI in primary care [104].

Immunoprophylactic approaches represent a promising frontier in recurrent UTI prevention.

Currently, OM-89 (Uro-Vaxom) is an oral immunostimulant composed of lyophilized lysates from multiple strains of *Escherichia coli*, used for the prophylaxis of recurrent urinary tract infections through modulation of host immune responses. A systematic review found OM-89 reduced UTI recurrence, with the maximal effect at 3 months post-treatment [105].

Also, there is MV140 (Uromune), a sublingual polybacterial vaccine that consists of heat-inactivated whole-cell preparations of common uropathogens, including *Escherichia coli*, *Klebsiella pneumoniae*, *Proteus vulgaris*, and *Enterococcus faecalis*, and it is used as an immunoprophylactic agent to reduce the incidence of recurrent urinary tract infections. Studies in frail elderly patients demonstrated a 7- to 40-fold reduction in UTI episodes following a 3-month course [106]. A retrospective study showed UTI episodes decreased from 3.73 to 0.98 per year (*p* < 0.001) [107].

Bacteriophage therapy could be an alternative for multidrug-resistant UTIs. Current evidence for bacteriophage therapy in UTIs remains limited but promising. A broad systematic review of phage therapy in difficult-to-treat infections identified 59 clinical studies, of which only seven addressed urological indications. Across all indications, clinical improvement was reported in 79% of patients and bacterial eradication in 87%, with adverse events in 7% of phage-treated patients compared with 15% of controls [108]. These figures should be interpreted with caution, since 58% of patients received concomitant antibiotics, which makes the independent contribution of phage therapy difficult to establish [108]. A subsequent systematic review restricted to urological indications, which evaluated these seven studies together with a further 38 reports, reached broadly similar conclusions; reported routes of administration include intravesical, oral, and intrarectal delivery [109].

The LBP-EC01 (ELIMINATE Trial) is a CRISPR-Cas3-enhanced bacteriophage cocktail that is under investigation for uncomplicated UTI caused by *E. coli.* This represents the first interventional study of a genetically enhanced phage cocktail, designed to maximize bactericidal properties and host range [110]. Phage-based therapies, particularly phage cocktails, have demonstrated efficacy in reducing UPEC colonization in experimental mouse models of bladder and kidney infection. In experimental models, bacteriophages have also been shown to inhibit biofilm formation and reduce bacterial adherence to uroepithelial cells, indicating a potential role in disrupting key virulence mechanisms [111,112].

**Figure 5:**
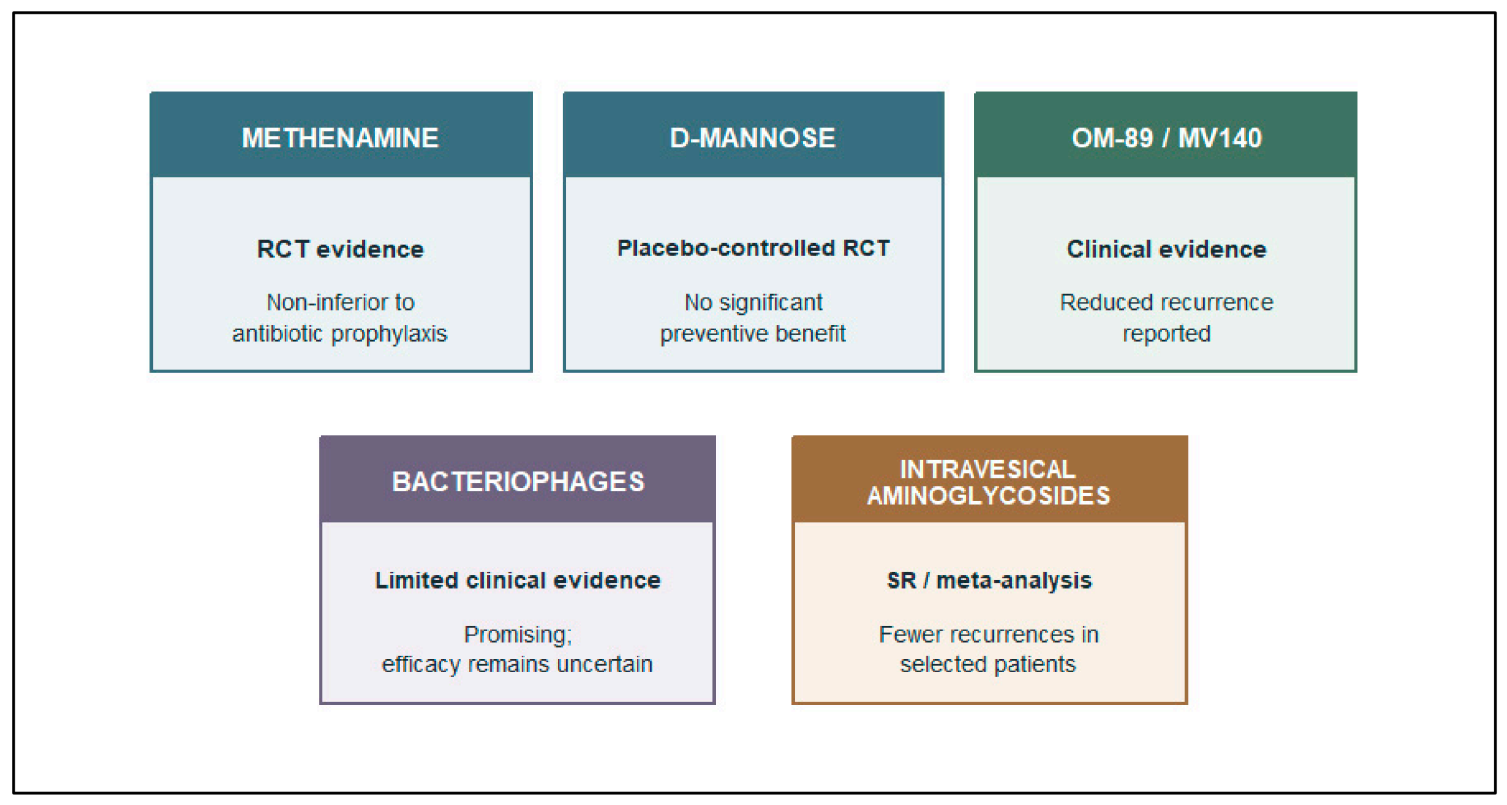
Emerging and adjunctive strategies for recurrent urinary tract infections.

Intravesical therapies have been a cornerstone of local treatment of UTIs. They provide localized, high-concentration treatment with minimal systemic absorption. For example, a systematic review and meta-analysis demonstrated that intravesical aminoglycoside therapy is associated with a substantial reduction in UTI recurrence [113]. In that analysis, the mean number of UTI episodes decreased from 4.8 to 1.0 during treatment [113]. In a separate cohort, a decline in antimicrobial resistance rates among uropathogens was observed, from 78% to 23% [114]. In a prospective single-centre study, intravesical gentamicin was not associated with detectable systemic absorption, and no clinically significant adverse effects were attributed to the treatment, although minor local effects have been reported in comparable series [115]. Treatment success rates of approximately 80% have been reported in a systematic review of intravesical aminoglycoside therapy [116]. A summary of these strategies is shown in Figure 5.

Research in this field remains dynamic and rapidly evolving, with ongoing investigations expected to yield novel diagnostic and therapeutic strategies. In particular, the integration of artificial intelligence is anticipated to play an increasingly transformative role, facilitating advanced data analysis, predictive modelling, and the development of personalized, precision-based approaches to disease management.

## 9. Conclusions and Future Directions

Urinary tract infections represent a complex and evolving clinical entity, now understood not merely as isolated microbial invasion but as a multifactorial process involving microbial dysbiosis, host–pathogen interactions, and environmental influences. The high rates of uropathogenic *Escherichia coli*, particularly virulent phylogroups such as B2, underscore the importance of specific virulence mechanisms including adhesion, intracellular bacterial community formation, and biofilm development in persistence and recurrence. Concurrently, the increasing prevalence of multidrug-resistant organisms, especially among ESKAPE pathogens, poses a significant global challenge, complicating treatment and highlighting the urgent need for effective antimicrobial stewardship and novel therapeutic strategies.

Advances in diagnostic approaches and updated clinical classifications have improved the precision of UTI management, emphasizing disease localization and clinical severity rather than traditional host-based definitions. Emerging therapies, including immunoprophylaxis, bacteriophage-based interventions, intravesical treatments, and microbiome-modulating strategies, offer promising alternatives to conventional antibiotics, particularly in the context of recurrent and resistant infections.

Future research should focus on integrating molecular insights with clinical practice, with particular emphasis on personalized medicine approaches. The application of artificial intelligence to large-scale microbiological and clinical datasets is expected to enhance predictive diagnostics, optimize treatment selection, and guide antimicrobial stewardship. Ultimately, a multidisciplinary and precision-based approach will be essential to address the growing burden and complexity of UTIs.

## Data Availability

Further information concerning the present study is available from the corresponding authors upon reasonable request.

## References

[1] Murray B.O., Flores C., Williams C., Flusberg D.A., Marr E.E., Kwiatkowska K.M., Charest J.L., Isenberg B.C., Rohn J.L. (2021). Recurrent Urinary Tract Infection: A Mystery in Search of Better Model Systems. Front. Cell. Infect. Microbiol..

[2] Aragón I.M., Herrera-Imbroda B., Queipo-Ortuño M.I., Castillo E., Del Moral J.S., Gómez-Millán J., Yucel G., Lara M.F. (2018). The Urinary Tract Microbiome in Health and Disease. Eur. Urol. Focus.

[3] Naji A., Siskin D., Woodworth M.H., Lee J.R., Kraft C.S., Mehta N. (2024). The Role of the Gut, Urine, and Vaginal Microbiomes in the Pathogenesis of Urinary Tract Infection in Women and Consideration of Microbiome Therapeutics. Open Forum Infect. Dis..

[4] Klein R.D., Hultgren S.J. (2020). Urinary tract infections: Microbial pathogenesis, host-pathogen interactions and new treatment strategies. Nat. Rev. Microbiol..

[5] Chieng C.C.Y., Kong Q., Liou N.S.Y., Khasriya R., Horsley H. (2023). The clinical implications of bacterial pathogenesis and mucosal immunity in chronic urinary tract infection. Mucosal Immunol..

[6] Flores-Mireles A.L., Walker J.N., Caparon M., Hultgren S.J. (2015). Urinary tract infections: Epidemiology, mechanisms of infection and treatment options. Nat. Rev. Microbiol..

[7] Terlizzi M.E., Gribaudo G., Maffei M.E. (2017). UroPathogenic *Escherichia coli* (UPEC) Infections: Virulence Factors, Bladder Responses, Antibiotic, and Non-antibiotic Antimicrobial Strategies. Front. Microbiol..

[8] García-García J.D., Contreras-Alvarado L.M., Cruz-Córdova A., Hernández-Castro R., Flores-Encarnacion M., Rivera-Gutiérrez S., Arellano-Galindo J.A., Ochoa S., Xicohtencatl-Cortes J. (2025). Pathogenesis and Immunomodulation of Urinary Tract Infections Caused by Uropathogenic *Escherichia coli*. Microorganisms.

[9] Johnson J.R. (1991). Virulence factors in *Escherichia coli* urinary tract infection. Clin. Microbiol. Rev..

[10] Le T., Wang L., Zeng C., Fu L., Liu Z., Hu J. (2021). Clinical and microbiological characteristics of nosocomial, healthcare-associated, and community-acquired *Klebsiella pneumoniae* infections in Guangzhou, China. Antimicrob. Resist. Infect. Control.

[11] Monteiro A.S.S., Silva M.O., Galvão V.S., Bomfim A.P., de Araújo L.G., Silva C.M.P., Barberino M.G., Gouveia E.L., Cordeiro S.M., Reis J.N. (2025). High proportions of multidrug-resistant *Klebsiella pneumoniae* isolates in community-acquired infections, Brazil. Sci. Rep..

[12] Fitzgerald M.J., Pearson M.M., Mobley H.L.T. (2024). *Proteus mirabilis* UreR coordinates cellular functions required for urease activity. J. Bacteriol..

[13] Johnson D.E., Russell R.G., Lockatell C.V., Zulty J.C., Warren J.W., Mobley H.L. (1993). Contribution of *Proteus mirabilis* urease to persistence, urolithiasis, and acute pyelonephritis in a mouse model of ascending urinary tract infection. Infect. Immun..

[14] Ch’ng J.H., Chong K.K.L., Lam L.N., Wong J.J., Kline K.A. (2019). Biofilm-associated infection by enterococci. Nat. Rev. Microbiol..

[15] Shakhatreh M.A.K., Rabaiah H.H., Khabour O.F., Jacob J.H., Alzoubi K.H. (2025). Detection of Virulence Factors and Antimicrobial Susceptibility among Clinical Strains of *Enterococcus faecalis*. Curr. Pharm. Biotechnol..

[16] Eriksson A., Giske C.G., Ternhag A. (2013). The relative importance of *Staphylococcus saprophyticus* as a urinary tract pathogen: Distribution of bacteria among urinary samples analysed during 1 year at a major Swedish laboratory. APMIS.

[17] Jordan P.A., Iravani A., Richard G.A., Baer H. (1980). Urinary tract infection caused by *Staphylococcus saprophyticus*. J. Infect. Dis..

[18] Smithson A., Ramos J., Bastida M.T., Bernal S., Jove N., Niño E., Msabri N., Porrón R. (2015). Differential characteristics of healthcare-associated compared to community-acquired febrile urinary tract infections in males. Eur. J. Clin. Microbiol. Infect. Dis..

[19] Okeke I.N., de Kraker M.E.A., Van Boeckel T.P., Kumar C.K., Schmitt H., Gales A.C., Bertagnolio S., Sharland M., Laxminarayan R. (2024). The scope of the antimicrobial resistance challenge. Lancet.

[20] Jiménez-Guerra G., Heras-Cañas V., Gutiérrez-Soto M., Del Pilar Aznarte-Padial M., Expósito-Ruiz M., Navarro-Marí J.M., Gutiérrez-Fernández J. (2018). Urinary tract infection by *Acinetobacter baumannii* and *Pseudomonas aeruginosa*: Evolution of antimicrobial resistance and therapeutic alternatives. J. Med. Microbiol..

[21] La Bella A.A., Andersen M.J., Gervais N.C., Molina J.J., Molesan A., Stuckey P.V., Wensing L., Nobile C.J., Shapiro R.S., Santiago-Tirado F.H. (2023). The catheterized bladder environment promotes Efg1- and Als1-dependent Candida albicans infection. Sci. Adv..

[22] Miller W.R., Arias C.A. (2024). ESKAPE pathogens: Antimicrobial resistance, epidemiology, clinical impact and therapeutics. Nat. Rev. Microbiol..

[23] Aloke C., Achilonu I. (2023). Coping with the ESKAPE pathogens: Evolving strategies, challenges and future prospects. Microb. Pathog..

[24] De Oliveira D.M.P., Forde B.M., Kidd T.J., Harris P.N.A., Schembri M.A., Beatson S.A., Paterson D.L., Walker M.J. (2020). Antimicrobial Resistance in ESKAPE Pathogens. Clin. Microbiol. Rev..

[25] Mulvey M.A., Lopez-Boado Y.S., Wilson C.L., Roth R., Parks W.C., Heuser J., Hultgren S.J. (1998). Induction and Evasion of Host Defenses by Type 1-Piliated Uropathogenic *Escherichia coli*. Science.

[26] Sauer M.M., Jakob R.P., Eras J., Baday S., Eriş D., Navarra G., Bernèche S., Ernst B., Maier T., Glockshuber R. (2016). Catch-bond mechanism of the bacterial adhesin FimH. Nat. Commun..

[27] Sokurenko E.V., Chesnokova V., Dykhuizen D.E., Ofek I., Wu X.-R., Krogfelt K.A., Struve C., Schembri M.A., Hasty D.L. (1998). Pathogenic adaptation of *Escherichia coli* by natural variation of the FimH adhesin. Proc. Natl. Acad. Sci. USA.

[28] Strömberg N., Marklund B., Lund B., Ilver D., Hamers A., Gaastra W., Karlsson K., Normark S. (1990). Host-specificity of uropathogenic Escherichia coli depends on differences in binding specificity to Gal alpha 1-4Gal-containing isoreceptors. EMBO J..

[29] Kudinha T., Kong F. (2022). Distribution of papG alleles among uropathogenic Escherichia coli from reproductive age women. J. Biomed. Sci..

[30] Flores-Mireles A.L., Pinkner J.S., Caparon M.G., Hultgren S.J. (2014). EbpA vaccine antibodies block binding of *Enterococcus faecalis* to fibrinogen to prevent catheter-associated bladder infection in mice. Sci. Transl. Med..

[31] Uhlén P., Laestadius A., Jahnukainen T., Söderblom T., Bäckhed F., Celsi G., Brismar H., Normark S., Aperia A., Richter-Dahlfors A. (2000). Alpha-haemolysin of uropathogenic *E. coli* induces Ca^2+^ oscillations in renal epithelial cells. Nature.

[32] Dhakal B.K., Mulvey M.A. (2012). The UPEC pore-forming toxin α-hemolysin triggers proteolysis of host proteins to disrupt cell adhesion, inflammatory, and survival pathways. Cell Host Microbe.

[33] Schmidt G., Sehr P., Wilm M., Selzer J., Mann M., Aktories K. (1997). Gln 63 of Rho is deamidated by *Escherichia coli* cytotoxic necrotizing factor-1. Nature.

[34] Flo T.H., Smith K.D., Sato S., Rodriguez D.J., Holmes M.A., Strong R.K., Akira S., Aderem A. (2004). Lipocalin 2 mediates an innate immune response to bacterial infection by sequestrating iron. Nature.

[35] Fischbach M.A., Lin H., Zhou L., Yu Y., Abergel R.J., Liu D.R., Raymond K.N., Wanner B.L., Strong R.K., Walsh C.T. (2006). The pathogen-associated iroA gene cluster mediates bacterial evasion of lipocalin 2. Proc. Natl. Acad. Sci. USA.

[36] Chaturvedi K.S., Hung C.S., Crowley J.R., Stapleton A.E., Henderson J.P. (2012). The siderophore yersiniabactin binds copper to protect pathogens during infection. Nat. Chem. Biol..

[37] Bates J.M., Raffi H.M., Prasadan K., Mascarenhas R., Laszik Z., Maeda N., Hultgren S.J., Kumar S. (2004). Tamm-Horsfall protein knockout mice are more prone to urinary tract infection: Rapid communication. Kidney Int..

[38] Song J., Bishop B.L., Li G., Grady R., Stapleton A., Abraham S.N. (2009). TLR4-mediated expulsion of bacteria from infected bladder epithelial cells. Proc. Natl. Acad. Sci. USA.

[39] Chromek M., Slamová Z., Bergman P., Kovács L., Podracká L., Ehrén I., Hökfelt T., Gudmundsson G.H., Gallo R.L., Agerberth B. (2006). The antimicrobial peptide cathelicidin protects the urinary tract against invasive bacterial infection. Nat. Med..

[40] Spencer J.D., Schwaderer A.L., Dirosario J.D., McHugh K.M., McGillivary G., Justice S.S., Carpenter A.R., Baker P.B., Harder J., Hains D.S. (2011). Ribonuclease 7 is a potent antimicrobial peptide within the human urinary tract. Kidney Int..

[41] Valore E.V., Park C.H., Quayle A.J., Wiles K.R., McCray P.B., Ganz T. (1998). Human beta-defensin-1: An antimicrobial peptide of urogenital tissues. J. Clin. Investig..

[42] Li X., Jiang L., Zhang S., Zhou J., Liu L., Jin C., Sun H., Wang Q., Liu Y., Pang Y. (2024). Uropathogenic *Escherichia coli* Subverts Host Autophagic Defenses by Stalling Preautophagosomal Structures to Escape Lysosome Exocytosis. J. Infect. Dis..

[43] Duraiswamy S., Chee J.L.Y., Chen S., Yang E., Lees K., Chen S.L. (2018). Purification of Intracellular Bacterial Communities during Experimental Urinary Tract Infection Reveals an Abundant and Viable Bacterial Reservoir. Infect. Immun..

[44] Justice S.S., Hung C., Theriot J.A., Fletcher D.A., Anderson G.G., Footer M.J., Hultgren S.J. (2004). Differentiation and developmental pathways of uropathogenic *Escherichia coli* in urinary tract pathogenesis. Proc. Natl. Acad. Sci. USA.

[45] Justice S.S., Hunstad D.A., Seed P.C., Hultgren S.J. (2006). Filamentation by *Escherichia coli* subverts innate defenses during urinary tract infection. Proc. Natl. Acad. Sci. USA.

[46] Mysorekar I.U., Hultgren S.J. (2006). Mechanisms of uropathogenic *Escherichia coli* persistence and eradication from the urinary tract. Proc. Natl. Acad. Sci. USA.

[47] De Nisco N.J., Neugent M., Mull J., Chen L., Kuprasertkul A., de Souza Santos M., Palmer K.L., Zimmern P., Orth K. (2019). Direct Detection of Tissue-Resident Bacteria and Chronic Inflammation in the Bladder Wall of Postmenopausal Women with Recurrent Urinary Tract Infection. J. Mol. Biol..

[48] Kalu M., Jorth P., Wong-Beringer A. (2025). Comparison of phenotypic and genetic traits of ESBL-producing UPEC strains causing recurrent or single episode UTI in postmenopausal women. Ann. Clin. Microbiol. Antimicrob..

[49] Armbruster C.E., Mobley H.L. (2012). Merging mythology and morphology: The multifaceted lifestyle of *Proteus mirabilis*. Nat. Rev. Microbiol..

[50] Paczosa M.K., Mecsas J. (2016). *Klebsiella pneumoniae*: Going on the Offense with a Strong Defense. Microbiol. Mol. Biol. Rev..

[51] Du D., Wang Z., James N.R., Voss J.E., Klimont E., Ohene-Agyei T., Venter H., Chiu W., Luisi B.F. (2014). Structure of the AcrAB-TolC multidrug efflux pump. Nature.

[52] Martínez-Martínez L. (2008). Extended-spectrum beta-lactamases and the permeability barrier. Clin. Microbiol. Infect..

[53] Hung C., Zhou Y., Pinkner J.S., Dodson K.W., Crowley J.R., Heuser J., Chapman M.R., Hadjifrangiskou M., Henderson J.P., Hultgren S.J. (2013). *Escherichia coli* biofilms have an organized and complex extracellular matrix structure. mBio.

[54] Zhao X., Lv Y., Adam F.E.A., Xie Q., Wang B., Bai X., Wang X., Shan H., Wang X., Liu H. (2021). Comparison of Antimicrobial Resistance, Virulence Genes, Phylogroups, and Biofilm Formation of *Escherichia coli* Isolated from Intensive Farming and Free-Range Sheep. Front. Microbiol..

[55] Clermont O., Christenson J.K., Denamur E., Gordon D.M. (2013). The Clermont *Escherichia coli* phylo-typing method revisited: Improvement of specificity and detection of new phylo-groups. Environ. Microbiol. Rep..

[56] Clermont O., Bonacorsi S., Bingen E. (2004). Characterization of an Anonymous Molecular Marker Strongly Linked to *Escherichia coli* Strains Causing Neonatal Meningitis. J. Clin. Microbiol..

[57] Abram K., Udaondo Z., Bleker C., Wanchai V., Wassenaar T.M., Robeson M.S., Ussery D.W. (2021). Mash-based analyses of *Escherichia coli* genomes reveal 14 distinct phylogroups. Commun. Biol..

[58] Toval F., Köhler C.-D., Vogel U., Wagenlehner F., Mellmann A., Fruth A., Schmidt M.A., Karch H., Bielaszewska M., Dobrindt U. (2014). Characterization of *Escherichia coli* isolates from hospital inpatients or outpatients with urinary tract infection. J. Clin. Microbiol..

[59] Wang M.C., Fan Y.H., Zhang Y.Z., Bregente C.J.B., Lin W.H., Chen C.A., Lin T.P., Kao C.Y. (2023). Characterization of uropathogenic *Escherichia coli* phylogroups associated with antimicrobial resistance, virulence factor distribution, and virulence-related phenotypes. Infection, genetics and evolution. J. Mol. Epidemiol. Evol. Genet. Infect. Dis..

[60] Clermont O., Condamine B., Dion S., Gordon D.M., Denamur E. (2021). The E phylogroup of *Escherichia coli* is highly diverse and mimics the whole *E. coli* species population structure. Environ. Microbiol..

[61] Khairy R.M., Mohamed E.S., Abdel Ghany H.M., Abdelrahim S.S. (2019). Phylogenic classification and virulence genes profiles of uropathogenic *E. coli* and diarrhegenic *E. coli* strains isolated from community acquired infections. PLoS ONE.

[62] Clermont O., Dixit O.V.A., Vangchhia B., Condamine B., Dion S., Bridier-Nahmias A., Denamur E., Gordon D. (2019). Characterization and rapid identification of phylogroup G in *Escherichia coli*, a lineage with high virulence and antibiotic resistance potential. Environ. Microbiol..

[63] Hashemizadeh Z., Kalantar-Neyestanaki D., Mansouri S. (2017). Association between virulence profile, biofilm formation and phylogenetic groups of *Escherichia coli* causing urinary tract infection and the commensal gut microbiota: A comparative analysis. Microb. Pathog..

[64] Rezatofighi S.E., Mirzarazi M., Salehi M. (2021). Virulence genes and phylogenetic groups of uropathogenic *Escherichia coli* isolates from patients with urinary tract infection and uninfected control subjects: A case-control study. BMC Infect. Dis..

[65] Tourret J., Denamur E. (2016). Population Phylogenomics of Extraintestinal Pathogenic *Escherichia coli*. Microbiol. Spectr..

[66] Hyun M., Lee J.Y., Kim H.A. (2021). Differences of virulence factors, and antimicrobial susceptibility according to phylogenetic group in uropathogenic *Escherichia coli* strains isolated from Korean patients. Ann. Clin. Microbiol. Antimicrob..

[67] Maniam L., Vellasamy K.M., Ong T.A., Teh C.S.J., Jabar K.A., Mariappan V., Narayanan V., Vadivelu J., Pallath V. (2023). Genotypic characteristics of Uropathogenic *Escherichia coli* isolated from complicated urinary tract infection (cUTI) and asymptomatic bacteriuria-a relational analysis. PeerJ.

[68] Dadi B.R., Abebe T., Zhang L., Mihret A., Abebe W., Amogne W. (2020). Distribution of virulence genes and phylogenetics of uropathogenic *Escherichia coli* among urinary tract infection patients in Addis Ababa, Ethiopia. BMC Infect. Dis..

[69] Ejrnæs K., Stegger M., Reisner A., Ferry S., Monsen T., Holm S.E., Lundgren B., Frimodt-Møller N. (2011). Characteristics of *Escherichia coli* causing persistence or relapse of urinary tract infections: Phylogenetic groups, virulence factors and biofilm formation. Virulence.

[70] Bonkat G., Wagenlehner F., Kranz J. (2024). Keep it Simple: A Proposal for a New Definition of Uncomplicated and Complicated Urinary Tract Infections from the EAU Urological Infections Guidelines Panel. Eur. Urol..

[71] Infectious Diseases Society of America (2025). Clinical Practice Guideline for the Management of Complicated Urinary Tract Infections.

[72] Hooton T.M. (2012). Clinical practice. Uncomplicated urinary tract infection. N. Engl. J. Med..

[73] Johnson J.R., Russo T.A. (2018). Acute Pyelonephritis in Adults. N. Engl. J. Med..

[74] Silverman M.D., Turrentine M.A. (2023). Urinary Tract Infections in Pregnant Individuals. Obstet. Gynecol..

[75] Geerlings S.E. (2016). Clinical Presentations and Epidemiology of Urinary Tract Infections. Microbiol. Spectr..

[76] Bjerklund Johansen T.E., Bahrs C., Bruyere F., Cai T., Joseph A., Köves B., Livermore D.M., Oliva A., Soriano A., Wagenlehner F. (2025). Consensus position statement on advancing the classification of patients and tests of cure in studies of antibiotic treatment of complicated urinary tract infections. Lancet. Infect. Dis..

[77] Heyns C.F. (2012). Urinary tract infection associated with conditions causing urinary tract obstruction and stasis, excluding urolithiasis and neuropathic bladder. World J. Urol..

[78] Hickling D.R., Sun T.T., Wu X.R. (2015). Anatomy and Physiology of the Urinary Tract: Relation to Host Defense and Microbial Infection. Microbiol. Spectr..

[79] Kamei J., Fujimura T. (2023). Urinary tract infection in patients with lower urinary tract dysfunction. J. Infect. Chemother..

[80] Ackerson B.K., Tartof S.Y., Chen L.H., Contreras R., Reyes I.A.C., Ku J.H., Pellegrini M., Schmidt J.E., Bruxvoort K.J. (2024). Risk Factors for Recurrent Urinary Tract Infections Among Women in a Large Integrated Health Care Organization in the United States. J. Infect. Dis..

[81] Arnold J.J., Hehn L.E., Klein D.A. (2016). Common Questions About Recurrent Urinary Tract Infections in Women. Am. Fam. Physician.

[82] Blango M.G., Ott E.M., Erman A., Veranic P., Mulvey M.A. (2014). Forced resurgence and targeting of intracellular uropathogenic *Escherichia coli* reservoirs. PLoS ONE.

[83] Mody L., Juthani-Mehta M. (2014). Urinary tract infections in older women: A clinical review. JAMA.

[84] Luu T., Albarillo F.S. (2022). Asymptomatic Bacteriuria: Prevalence, Diagnosis, Management, and Current Antimicrobial Stewardship Implementations. Am. J. Med..

[85] Henderson J.T., Webber E.M., Bean S.I. (2019). Screening for Asymptomatic Bacteriuria in Adults: Updated Evidence Report and Systematic Review for the US Preventive Services Task Force. JAMA.

[86] Daniel M., Keller S., Mozafarihashjin M., Pahwa A., Soong C. (2018). An Implementation Guide to Reducing Overtreatment of Asymptomatic Bacteriuria. JAMA Intern. Med..

[87] Wiley Z., Jacob J.T., Burd E.M. (2020). Targeting Asymptomatic Bacteriuria in Antimicrobial Stewardship: The Role of the Microbiology Laboratory. J. Clin. Microbiol..

[88] Chu C.M., Lowder J.L. (2018). Diagnosis and treatment of urinary tract infections across age groups. Am. J. Obstet. Gynecol..

[89] Moragas A., Monfà R., García-Sangenís A., Llor C. (2026). Accuracy of leukocyte esterase and nitrite tests for diagnosing bacteriuria in older adults: A systematic review and meta-analysis. Clin. Microbiol. Infect..

[90] Veauthier B., Miller M.V. (2020). Urinary Tract Infections in Young Children and Infants: Common Questions and Answers. Am. Fam. Physician.

[91] Miller J.M., Binnicker M.J., Campbell S., Carroll K.C., Chapin K.C., Gilligan P.H., Gonzalez M.D., Jerris R.C., Kehl S.C., Patel R. (2018). A guide to utilization of the microbiology laboratory for diagnosis of infectious diseases: 2018 update by the Infectious Diseases Society of America and the American Society for Microbiology. Clin. Infect. Dis..

[92] Kurotschka P.K., Gágyor I., Ebell M.H. (2024). Acute Uncomplicated UTIs in Adults: Rapid Evidence Review. Am. Fam. Physician.

[93] Smith A.D., Nikolaidis P., Khatri G., Chong S.T., De Leon A.D., Ganeshan D., Gore J.L., Gupta R.T., Kwun R., Lyshchik A. (2022). ACR Appropriateness Criteria^®^ Acute Pyelonephritis: 2022 Update. J. Am. Coll. Radiol..

[94] Lee R.A., Centor R.M., Humphrey L.L., Jokela J.A., Andrews R., Qaseem A. (2021). Appropriate Use of Short-Course Antibiotics in Common Infections: Best Practice Advice from the American College of Physicians. Ann. Intern. Med..

[95] Kim D.K., Kim J.H., Lee J.Y., Ku N.S., Lee H.S., Park J.Y., Kim J.W., Kim K.J., Cho K.S. (2020). Reappraisal of the treatment duration of antibiotic regimens for acute uncomplicated cystitis in adult women: A systematic review and network meta-analysis of 61 randomised clinical trials. Lancet Infect. Dis..

[96] Gupta K., Hooton T.M., Naber K.G., Wullt B., Colgan R., Miller L.G., Moran G.J., Nicolle L.E., Raz R., Schaeffer A.J. (2011). International clinical practice guidelines for the treatment of acute uncomplicated cystitis and pyelonephritis in women: A 2010 update by the Infectious Diseases Society of America and the European Society for Microbiology and Infectious Diseases. Clin. Infect. Dis..

[97] Bassetti M., Echols R., Matsunaga Y., Ariyasu M., Doi Y., Ferrer R., Lodise T.P., Naas T., Niki Y., Paterson D.L. (2021). Efficacy and safety of cefiderocol or best available therapy for the treatment of serious infections caused by carbapenem-resistant Gram-negative bacteria (CREDIBLE-CR): A randomised, open-label, multicentre, pathogen-focused, descriptive, phase 3 trial. Lancet Infect. Dis..

[98] Dakkak M., Sabharwal M. (2022). Antibiotic Courses for Common Infections: Recommendations from the ACP. Am. Fam. Physician.

[99] Sher E.K., Džidić-Krivić A., Sesar A., Farhat E.K., Čeliković A., Beća-Zećo M., Pinjic E., Sher F. (2024). Current state and novel outlook on prevention and treatment of rising antibiotic resistance in urinary tract infections. Pharmacol. Ther..

[100] US Food and Drug Administration (2026). Approved Drug Products with Therapeutic Equivalence Evaluations (Orange Book).

[101] Wagenlehner F., Perry C.R., Hooton T.M., E Scangarella-Oman N., Millns H., Powell M., Jarvis E., Dennison J., Sheets A., Butler D. (2024). Oral gepotidacin versus nitrofurantoin in patients with uncomplicated urinary tract infection (EAGLE-2 and EAGLE-3): Two randomised, controlled, double-blind, double-dummy, phase 3, non-inferiority trials. Lancet.

[102] Harding C., Mossop H., Homer T., Chadwick T., King W., Carnell S., Lecouturier J., Abouhajar A., Vale L., Watson G. (2022). Alternative to prophylactic antibiotics for the treatment of recurrent urinary tract infections in women: Multicentre, open label, randomised, non-inferiority trial. BMJ.

[103] Heltveit-Olsen S.R., Arnljots E.S., Sundvall P.-D., Gunnarsson R., Kowalczyk A., Godycki-Cwirko M., Platteel T.N., Groen W.G., Lithén S.S., Sundvall S. (2025). Methenamine hippurate as prophylaxis for recurrent urinary tract infections in older women-a triple-blind, randomised, placebo-controlled, phase IV trial (ImpresU). Clin. Microbiol. Infect..

[104] Hayward G., Mort S., Hay A.D., Moore M., Thomas N.P.B., Cook J., Robinson J., Williams N., Maeder N., Edeson R. (2024). d-Mannose for Prevention of Recurrent Urinary Tract Infection Among Women: A Randomized Clinical Trial. JAMA Intern. Med..

[105] Beerepoot M.A., Geerlings S.E., van Haarst E.P., van Charante N.M., ter Riet G. (2013). Nonantibiotic prophylaxis for recurrent urinary tract infections: A systematic review and meta-analysis of randomized controlled trials. J. Urol..

[106] Lorenzo-Gómez M.F., Padilla-Fernández B., Flores-Fraile J., Valverde-Martínez S., González-Casado I., Hernández J.-M.D.D., Sánchez-Escudero A., Arroyo M.-J.V., Martínez-Huélamo M., Criado F.H. (2021). Impact of whole-cell bacterial immunoprophylaxis in the management of recurrent urinary tract infections in the frail elderly. Vaccine.

[107] Iftimie S., Ladero-Palacio P., López-Azcona A.F., Pujol-Galarza L., Pont-Salvadó A., Gabaldó-Barrios X., Joven J., Camps J., Castro A., Pascual-Queralt M. (2025). Evaluating the use of Uromune^®^ autovaccine in recurrent urinary tract infections: A pilot unicenter retrospective study in Reus, Spain. BMC Infect. Dis..

[108] Uyttebroek S., Chen B., Onsea J., Ruythooren F., Debaveye Y., Devolder D., Spriet I., Depypere M., Wagemans J., Lavigne R. (2022). Safety and efficacy of phage therapy in difficult-to-treat infections: A systematic review. Lancet Infect. Dis..

[109] Al-Anany A.M., Hooey P.B., Cook J.D., Burrows L.L., Martyniuk J., Hynes A.P., German G.J. (2023). Phage Therapy in the Management of Urinary Tract Infections: A Comprehensive Systematic Review. PHAGE Ther. Appl. Res..

[110] Kim P., Sanchez A.M., Penke T.J.R., Tuson H.H., Kime J.C., McKee R.W., Slone W.L., Conley N.R., McMillan L.J., Prybol C.J. (2024). Safety, pharmacokinetics, and pharmacodynamics of LBP-EC01, a CRISPR-Cas3-enhanced bacteriophage cocktail, in uncomplicated urinary tract infections due to *Escherichia coli* (ELIMINATE): The randomised, open-label, first part of a two-part phase 2 trial. Lancet Infect. Dis..

[111] Mongkolkarvin P., Sukjoi C., Suyapoh W., Buddhasiri S., Ilugbusi I.E., Nonejuie P., Hsieh M.H., Chaikeeratisak V., Thiennimitr P. (2026). Cocktail of genetically diverse lytic phages reduces uropathogenic *Escherichia coli* colonization in mouse urinary tract. Sci. Rep..

[112] González-Villalobos E., Ribas-Aparicio R.M., Montealegre G.E.R., Belmont-Monroy L., Ortega-García Y., Aparicio-Ozores G., Balcázar J.L., Eslava-Campos C.A., Hernández-Chiñas U., Molina-López J. (2021). |Isolation and characterization of novel bacteriophages as a potential therapeutic option for *Escherichia coli* urinary tract infections. Appl. Microbiol. Biotechnol..

[113] Kwon M., Ahmad A., Lott N., Blatt A. (2026). Intravesical therapy for recurrent urinary tract infection: A systematic review and meta-analysis. BJU Int..

[114] Stalenhoef J.E., van Nieuwkoop C., Menken P.H., Bernards S.T., Elzevier H.W., van Dissel J.T. (2019). Intravesical Gentamicin Treatment for Recurrent Urinary Tract Infections Caused by Multidrug Resistant Bacteria. J. Urol..

[115] Kinnear N., Coleman L., Parsons K., Casey L., Baverstock R. (2026). Safety and Efficacy of Intravesical Gentamicin for Recurrent Urinary Tract Infections and/or Catheter Blockages. Neurourol. Urodyn..

[116] Ong A., Pietropaolo A., Brown G., Somani B.K. (2022). Are Intravesical Aminoglycosides the New Gold Standard in the Management of Refractory Urinary Tract Infection: A Systematic Review of Literature. J. Clin. Med..

